# Simultaneous assessment of cell morphology and adhesion using aluminum nanoslit-based plasmonic biosensing chips

**DOI:** 10.1038/s41598-019-43442-w

**Published:** 2019-05-10

**Authors:** Hsien-San Hou, Kuang-Li Lee, Chen-Hung Wang, Tung-Han Hsieh, Juan-Jie Sun, Pei-Kuen Wei, Ji-Yen Cheng

**Affiliations:** 1 Research Center for Applied Sciences, Academia Sinica, Taipei, 11529 Taiwan; 2 Institute of Biophotonics, National Yang-Ming University, Taipei, 11221 Taiwan; 3 Department of Mechanical and Mechatronic Engineering, National Taiwan Ocean University, Keelung, 20224 Taiwan; 4 College of Engineering, Chang Gung University, Taoyuan, 33302 Taiwan

**Keywords:** Nanoscience and technology, Characterization and analytical techniques, Biological techniques, Analytical chemistry

## Abstract

A variety of physiological and pathological processes rely on cell adhesion, which is most often tracked by changes in cellular morphology. We previously reported a novel gold nanoslit-based biosensor that is capable of real-time and label-free monitoring of cell morphological changes and cell viability. However, the preparation of gold biosensors is inefficient, complicated and costly. Recently, nanostructure-based aluminum (Al) sensors have been introduced for biosensing applications. The Al-based sensor has a longer decay length and is capable of analyzing large-sized mass such as cells. Here, we developed two types of double-layer Al nanoslit-based plasmonic biosensors, which were nanofabricated and used to evaluate the correlation between metastatic potency and adhesion of lung cancer and melanoma cell lines. Cell adhesion was determined by Fano resonance signals that were induced by binding of the cells to the nanoslit. The peak and dip of the Fano resonance spectrum respectively reflected long- and short-range cellular changes, allowing us to simultaneously detect and distinguish between focal adhesion and cell spreading. Also, the Al nanoslit-based biosensor chips were used to evaluate the inhibitory effects of drugs on cancer cell spreading. We are the first to report the use of double layer Al nanoslit-based biosensors for detection of cell behavior, and such devices may become powerful tools for anti-metastasis drug screening in the future.

## Introduction

Cell adhesion is involved in a variety of physiological and pathological responses, such as cell differentiation, immune response, inflammation, embryogenesis, and tumor metastasis^1–4^. The process of cell adhesion can be divided into three steps, which include cell-substrate contact, cell spreading and cytoskeleton reorganization^5,6^. During the process of cell adhesion, the main observable change is the morphological transition of cells from spherical to flat. The surface membrane proteins involved in adhesion are called cell adhesion molecules (CAMs) and are classified as integrins, immunoglobulins, lymphocyte homing receptors, cadherins and selectins^7^. These CAMs are responsible for the specificity of intracellular binding as well as binding of cells to the extracellular matrix (ECM). As a result, selective binding allows a cell to adhere to a particular tissue, where it may perform essential cellular biological functions in the living organism. After CAMs bind to the ECM, tyrosine kinases such as Src and focal adhesion kinase (FAK) are recruited and activated, stimulating focal adhesion. Activated FAK then triggers the reorganization of α-actin and other structural cytoskeletal proteins, which induces the flattening of cell morphology and completion of cell adhesion^3,6,8^. The process of cell adhesion plays an essential role in cancer cell metastasis^4^, making CAMs potential targets for cancer therapy^9^. However, the influence of adhesion rate on the metastatic potency of cancer cells has not been fully elucidated. Thus, accurate tests of the correlation between metastatic efficacy and cancer cell adhesion may facilitate the development of new strategies for cancer treatment.

Several methods can be used to assess cell adhesion. In conventional adhesion assays, colorimetric^10^ or fluorometric^11^ detection methods have been used to quantify cell adhesion; however, the experimental approaches are time-consuming, insensitive and complicated. Cell morphological changes can also be detected using optical^12^ and electrical^13^ approaches. Prism-based surface plasmon resonance (SPR) optical sensing systems and electrical biosensors have both proven useful for analyzing cell adhesion^14,15^. Both methods are sensitive, but optical assays are less invasive to the cells because electrical stimulation can potentially activate FAK and influence cell adhesion^16^.

Localized SPR, generated by metallic nanostructures (e.g., nanoholes, nanorods, and nanoslits)^17^, and prism-based SPR, also known as conventional SPR, can both be used to perform real-time, label-free kinetic monitoring of biomolecules. Conventional SPR detection requires a glass prism and precise control of incident light angle^14^. Moreover, chip-based high-throughput and miniaturized systems are expensive and difficult to set up. In a conventional SPR system, cell morphology changes are detected via changes in the reflective intensity and angle of incident light through the prism. Therefore, the prism blocks the optical path needed for phase-contrast imaging. Because clear phase-contrast images of the test cells are not attainable with conventional SPR, it is not possible to precisely correlate cellular morphology responses and SPR signals from prism-based SPR sensors.

To overcome this limitation, we designed and developed a cell adhesion assessment system (CAAS) using an inverted microscope to gather phase-contrast images of the cells and carry out simultaneous surface plasmonic sensing. We used nanostructured SPR chips that allow cell attachment and measurement of transmitted light through the chip. The resonance changes in the transmission spectra were quantified and correlated to observed cell behaviors.

In the last two decades, an increasing number of studies have used localized SPR for biomolecule detection or cell behavior analysis^18^. Localized SPR can be generated by metal nanoparticles or periodic metal nanoslit arrays. Although gold is the most commonly used metal in localized SPR-based sensors, nanostructure-based aluminum (Al) sensors, such as nanoconcave arrays^19^, nanoholes^20^ and triangular nanoparticles^21^, have been developed because Al is a cost-effective plasmonic material that is useful for short-wavelength surface plasmons. Previously we developed a non-labeling optical method based on localized SPR on gold nanoslit (AuNS) array film to study the influence of anti-cancer drugs or fluidic shear stress on adhesion, detachment, and mortality of adherent cells^22,23^. However, the preparation of such AuNS biosensors is inefficient, complicated and costly. Recently, a sensitive double-layer capped Al nanoslit (CPALNS) biosensor has been used to detect biomolecules^24^. The double-layer nanoslit biosensor showed better sensitivity than monolayer nanoslit sensors^24^. In addition to the previous CPALNS sensor, we also fabricated a novel design with a complementary structure to the CPALNS biosensor, a double-layer grooved Al nanoslit (GOALNS) biosensor. The respective structures at the nanoslit sites of the CPALNS and the GOALNS biosensors are ridges and grooves. Here, we tested the capabilities of the two novel localized SPR-based biosensors to detect cell adhesion properties of a panel of cancer cells.

For nanoslit-based biosensors, the intensity change and the spectral shift of the Fano resonance dip/peak are used for molecule sensing^24,25^. In our study, we used resonance wavelength and intensity interrogation methods to evaluate cell adhesion kinetics. Specifically, the cell adhesion rate was determined by the time constant calculated according to the spectral intensity integration (d*A*), differential spectral peak intensity (d*I*), dip shift and peak shift of the Fano resonance signals. We then compared the adhesion rates among different types of cancer cells. It has been reported that inhibiting the activity of FAK in human melanoma^26^ or human non-small-cell lung cancer cells^27^ results in decreased metastatic capacity. In order to test for the correlations between metastatic potency and the adhesion rate among the cancer cells, metastatic human lung cancer and human melanoma cells were evaluated using the CPALNS and GOALNS biosensor chips. In addition, a cell adhesion inhibitor was applied to test whether the cell adhesion sensor chip can detect the effects of a drug on cell adhesion.

## Results and Discussion

### Optical setup of the CAAS and Al nanoslit-based plasmonic biosensors

**Figure 1:**
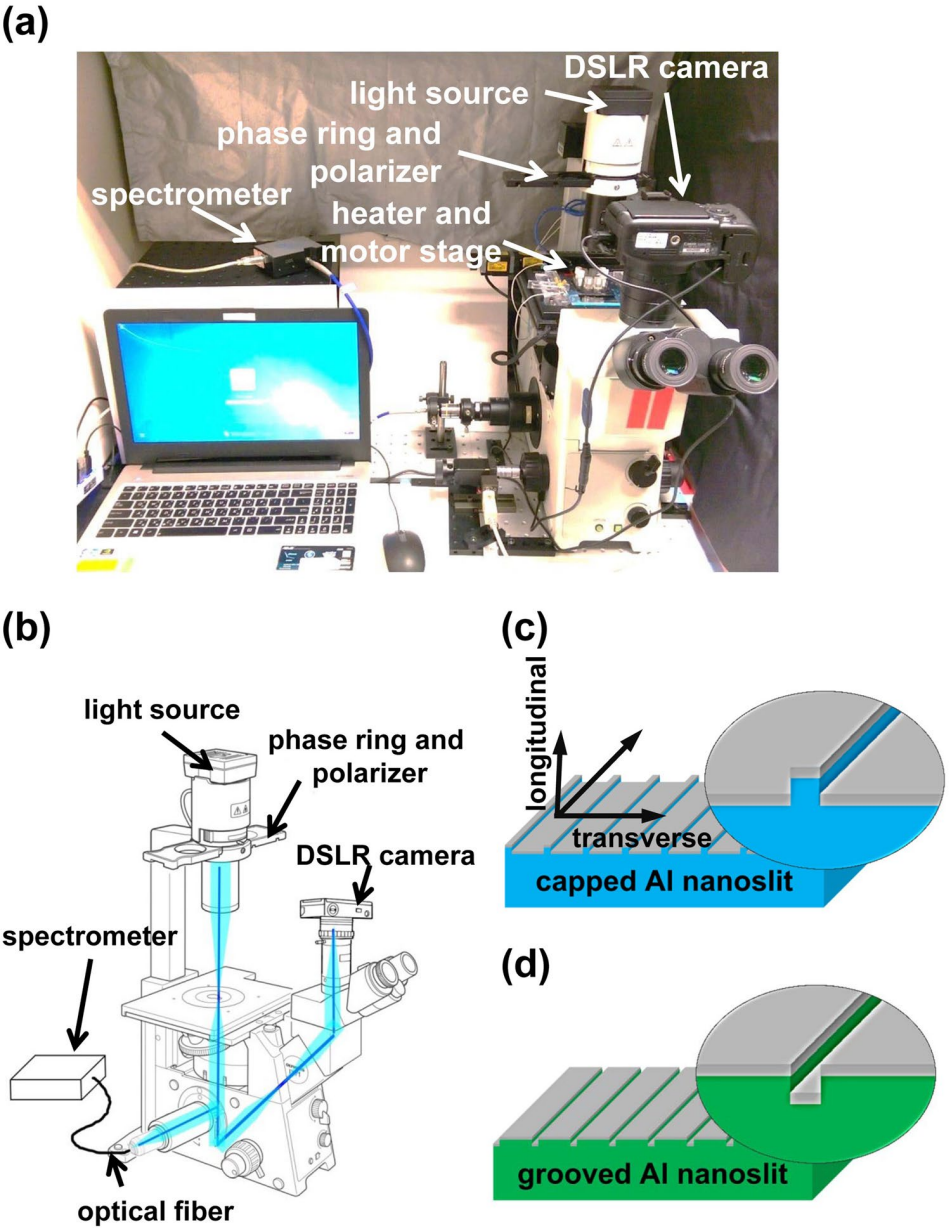
The optical setup and the nanoslit-based sensors for cell adhesion assessment. (**a**) A photograph of the setup shows the CAAS. The inverted microscope can detect cell morphological and spectral changes and is equipped with a motorized stage, camera, spectrometer, transparent heater and a computer control system. (**b**) (Modified from the microscope instruction manual) The optical path in the system is shown. The spectrum signal was detected from the side port of the microscope. The cell image was obtained from a camera mounted on the trinocular observation tube. The schematic diagram shows the (**c**) CPALNS and (**d**) GOALNS biosensors that were used for the cell adhesion sensing in current study. The grey layer indicates the deposition of Al on the nanofabricated PC film (blue) and plate (green).

**Figure 2:**
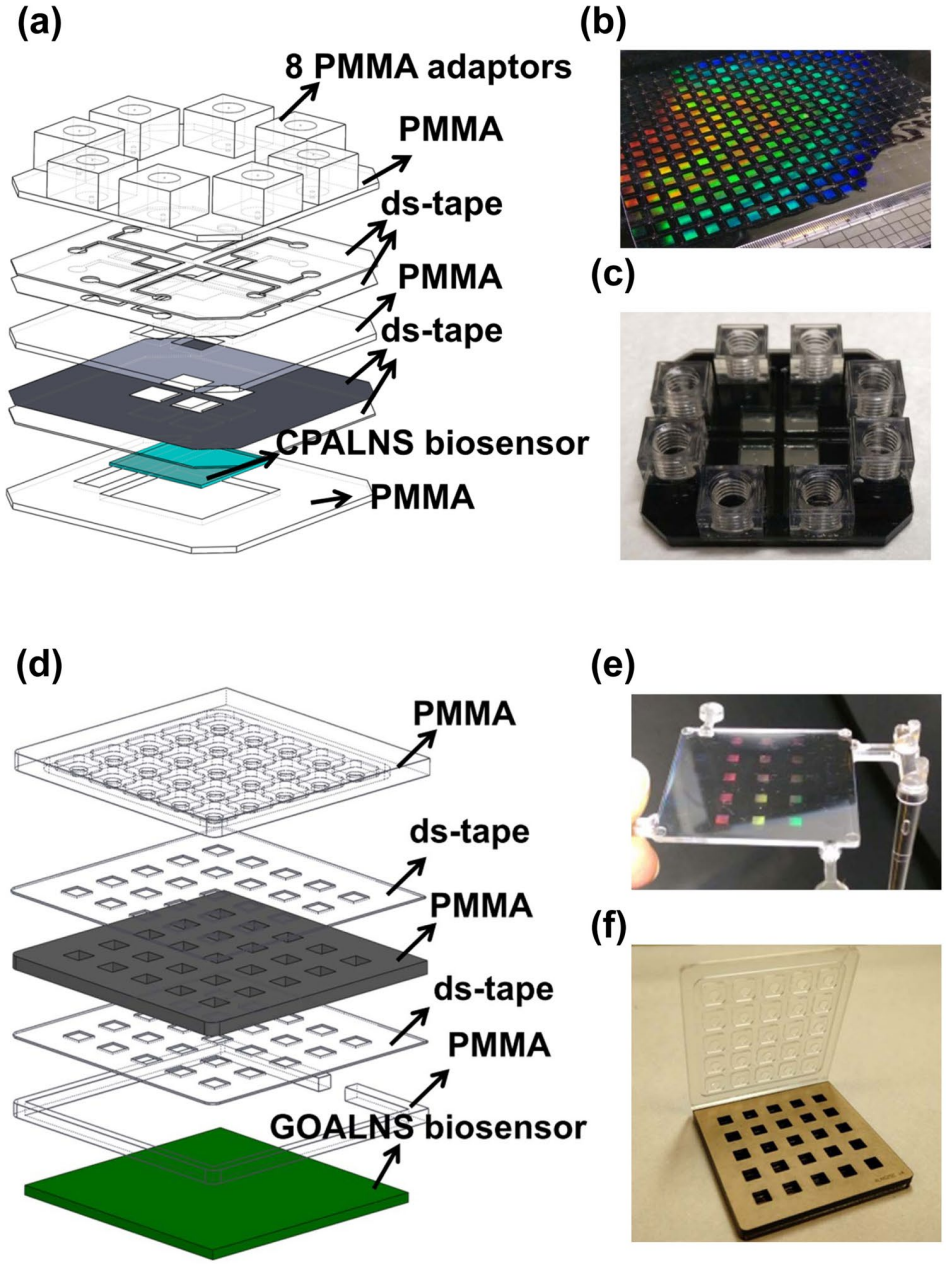
The fabrication of aluminum nanoslit-based biosensor chips for cell adhesion studies. (**a**) The design of the CPALNS4c chip. The CPALNS4c chip is composed of 8 PMMA adaptors, three layers of PMMA, four layers of double-sided tape and the CPALNS biosensor. (**b**) The photographs show the hot-embossing nanoimprinting polycarbonate film with nano-ridge arrays on the surface, and (**c**) the completed assembly of the CPALNS4c chip. (**d**) The design of the GOALNS25c chip. The GOALNS25c chip is composed of a PMMA cover, two layers of PMMA, double-sided tape and the GOALNS biosensor. (**e**) The photographs show the injection molding polycarbonate plate with nano-groove arrays on the surface, and (**f**) the complete assembly of the GOALNS25c chip.

The configurations of the CAAS and the Al nanoslit-based biosensor chips are shown in Figs 1 and 2. By using the CAAS and the localized SPR-based Al biosensors, we expected to be able to simultaneously observe cell phase-contrast images and perform surface plasmonic sensing during cell adhesion testing. The CPALNS biosensor was shown to be more sensitive for protein detection than an AuNS sensor^24^. Furthermore, a monolayer AuNS sensor has been used to detect cancer cells in human blood^28^. The monolayer AuNS sensor shown in Fig. S1 was made by a thermal-annealing template-stripping method^29^, which is complicated and time-consuming. Moreover, compared to the AuNS sensor, the preparation time and effort for the CPALNS and the GOALNS biosensor were greatly reduced by using hot-embossing nanoimprint and injection molding (please see Materials and Methods). Therefore, these biosensors are more suitable for mass production as a commercial product. In addition, using Al has an additional benefit over using Au.

It has been recently reported that long-range SPR with a longer decay length (500–1000 nm) is more sensitive than the conventional SPR (100–200 nm) and suitable for analysis of large-sized mass such as cells^30,31^. Compared to the gold-based SPR sensor, the Al-based SPR sensor has a longer decay length. For the surface plasmon wave propagating on a flat metal surface, the decay length *l*_*d*_ (where the amplitude drops to 1/e) is determined primarily by the resonance wavelength λ and can be expressed as follows^32^:

1 $${\iota }_{d}={\rm{Im}}\,{[\frac{\lambda }{2\pi }(\frac{{\varepsilon }_{d}+{\varepsilon }_{m}}{{\varepsilon }_{d}^{2}})]}^{1/2},$$

where *ε*_*m*_ and *ε*_*d*_ are the relative permittivities of metal and the adjacent dielectric material, the wavelength dependence permittivity of Al and Au are obtained from previous studies^33,34^. In Fig. S2, the calculated decay length at the wavelength of 470 nm for Al film is three folds longer than Au film. These studies suggested that Al nanoslit-based biosensors are more sensitive and suitable than the gold sensor for sensing a large mass analyte, such as cells.

### Design of the plasmonic biosensor chips for cell sensing

The CPALNS4c chip was designed to be used for cell sensing in a microfluidic system. A continuous-flow media supply system was connected to the CPALNS4c chip through the polymethylmethacrylate (PMMA) adaptors (Fig. 2c), thereby enabling long-term observation periods. As shown in Fig. 2f, the GOALNS25c chip was designed to have an open-well format. The well-to-well distance is 9 mm, which is compatible with that of 96-well microplates. Additionally, the cover lid was designed to prevent reagent cross-contamination between wells. Thus, the chip may be used with automated liquid handling systems for screening of drugs that modulate cell adhesion. These features for chip-based and high throughput label-free detection make the Al plasmonic biosensor chips better than conventional SPR-based biosensors.

### Optical properties of the nanoslit-based plasmonic biosensors

**Figure 3:**
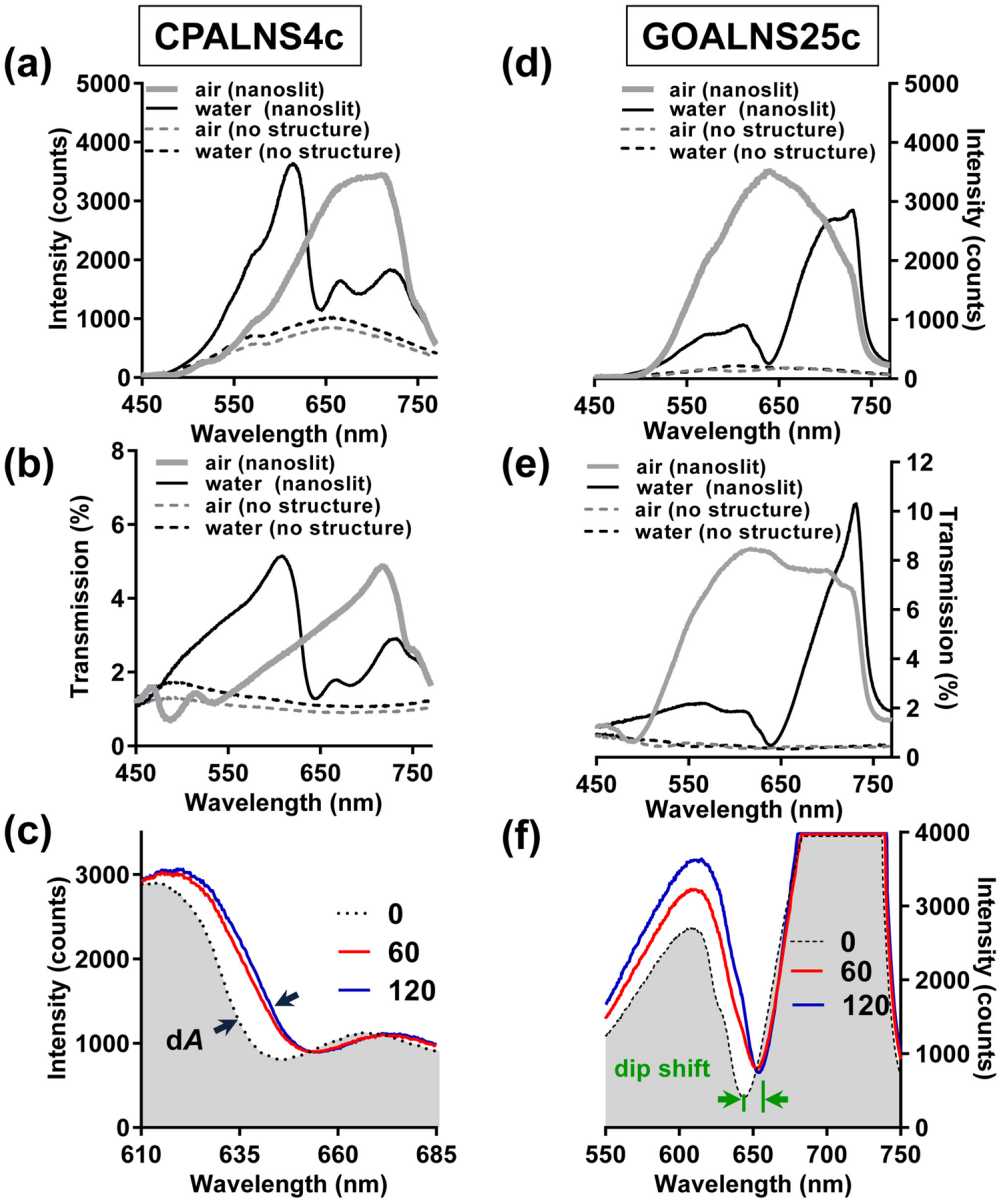
The optical properties of aluminum nanoslit-based biosensors. The optical properties of the double-layer (**a**–**c**) capped and (**d**–**f**) grooved Al nanoslit biosensors in the respective CPALNS4c and GOALNS25c chips. **(a,d**) The intensity spectra and (**b,e**) the transmission spectra of the Al biosensor chips under the water-filled or air-filled conditions. The intensity spectra shift of the Fano resonance induced by (**c**) A549 and (**f**) MDCK cell attachment and spreading at 0, 60 and 120 mins after cell seeding in the CPALNS4c and GOALNS25c chips, respectively.

Transmission spectra of the CPALNS4c chip (Fig. 3a,c) and the GOALNS25c chip (Fig. 3d,e) were measured using our CAAS. In the water-filled chamber, the intensity spectrum of the CPALNS4c chip showed a Fano resonance peak and dip at 615 nm and 645 nm, respectively (Fig. 3a,b). When the chambers were filled with air, we observed a peak at 468 nm (Fig. 3b), which is close to the expected wavelength of 470 nm^24^. For the GOALNS25c chip, specific and obvious dips were observed in the intensity spectrum and transmission spectrum when the chip was in contact with water. Although the transmission spectra represent the feature of the resonance of nanoslit sensors, we used the intensity spectra to analyze the kinetics of cell adhesion. The use of intensity spectra for the analysis simplified the process and the spectral difference could be observed while the artifact from the light source was subtracted. The Fano resonance spectrum of the Al nanoslit-based biosensor is comprised of the 3-mode coupling resonance of Cavity resonance, Wood’s anomaly and SPR^24^. In the previous study, Fano resonances could be easily modulated in CPALNS sensors by changing the ridge height of nanoslits and the deposited metal film thickness. Depending on the ridge height and the metal thickness, the transmission spectrum could range from a Wood’s anomaly-dominant resonance (peak) to an asymmetric Fano profile (peak and dip) or an SPR-dominant resonance (dip). Moreover, the differential wavelength shifts of the localized-SPR peak and dip are determined by the period of the nanoslit sensor^24^. In this study, the transmission spectrum indicates that the Fano resonance of the CPALNS biosensor is an asymmetric Fano profile (peak at 610 nm, dip at 644 nm) (Fig. 3b), while the GOALNS biosensor shows an SPR-dominant (dip at 638 nm) resonance (Fig. 3e).

The changes of the Fano resonance induced by the cell adhesion in the biosensor chips were further scrutinized. In the CPALNS4c chip, the Fano resonance exhibited a spectral redshift and intensity increase corresponding to the process of cell adhesion (Fig. 3c). The overall intensity changes together with the spectral shift were used to calculate d*A*, similar to the calculated parameter used for biomolecule sensing in previous studies^22,23,35^. The composite parameter d*A* was then correlated with the cell adhesion process. In the GOALNS25c chip, the intensity spectra shown in Fig. 3f revealed a significant redshift of the resonance during cell adhesion. Accordingly, the intensity of the resonance peak also increased with time during cell adhesion. As shown in Fig. 3c,f, the shifting in resonance dips stopped earlier than the intensity increasing of the resonance peaks in both Al biosensor chips (red line versus blue line). This result implied that the resonance dip and the peak of the Al nanoslit biosensor chips might sense different aspects of cellular behavior.

The sensors reported in this work could monitor molecular event in two different distance ranges. In our earlier study, the average calculated decay lengths were 338 nm for the resonance dip and 892 nm for the resonance peak in the CPALNS biosensor^24^. SPR signals generated by cell adhesion for two different types of sensors with different characteristic penetration depths were previously reported by Méjard *et al*.^36^ Conventional SPR utilizes a single layer of thin gold film for sensing and is characterized by single short penetration dimensions. Alternatively, long-range SPR, which uses an additional fluoropolymer polydecafluoroxaheptadiene (Cytop) layer on the thin gold film, is characterized by long penetration dimensions but still offering single range of penetration. The plasmonic resonance penetration depths of conventional SPR and long-range SPR are 100–200 nm and 500–1000 nm, respectively^36^. Notably, prism-based SPR sensors can only detect a single depth at one time. Our CPALNS biosensor is preferable over prism-based SPR sensors because it is capable of simultaneous sensing with dual penetration (or detection) depth. Similarly, using GOALNS chip, the dynamics of cell adhesion detected by the resonance dip are different from those by the resonance peak. Thus, the GOALNS biosensor can also simultaneously sense molecular events in dual penetration depths. As a comparison, resonant waveguide grating (RWG) biosensor is also capable of cell adhesion assessment^37^, however, the sensor can only detect a single dimension changes. Our biosensors are preferable to prism-based SPR and RWG sensors because they are capable of simultaneous dual detection depth sensing.

The main changes during cell adhesion are related to cell morphological reshaping in a two-dimensional plane and in the longitudinal direction. Since our biosensors produced different signal responses corresponding to long-range and short-range behaviors during the cell adhesion test, the data may provide useful information regarding both the cellular flattening and the extension in the longitudinal direction.

### Assessing cell adhesion by fano resonance and cell coverage changes using the CPALNS4c biosensor chip

**Figure 4:**
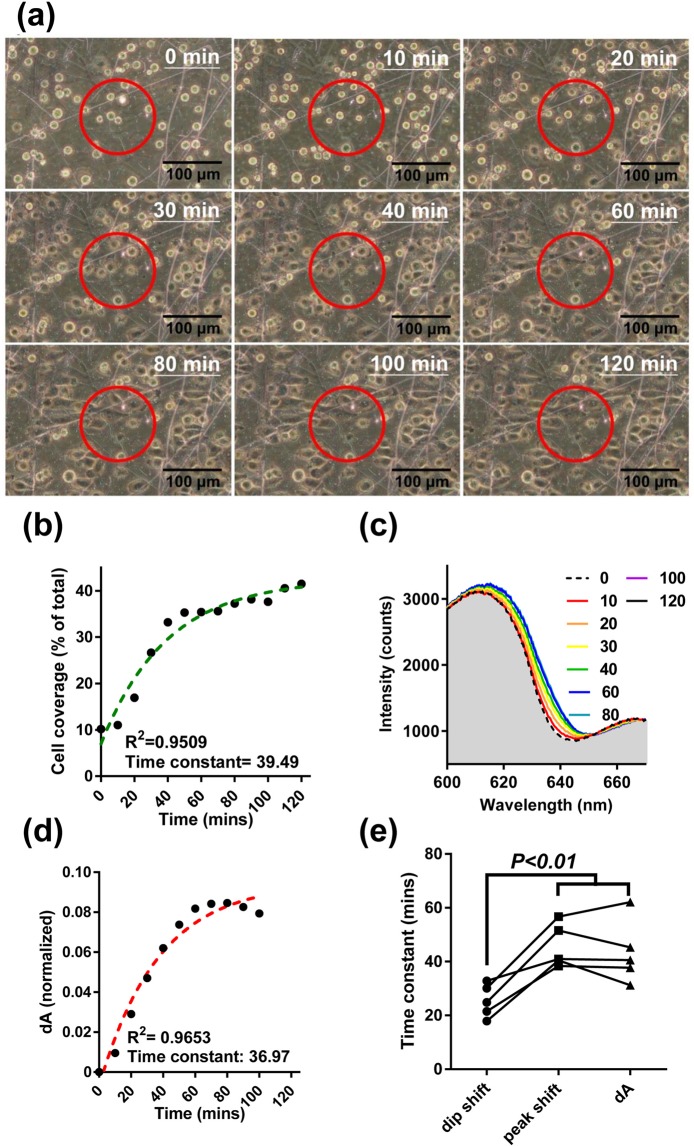
The Fano resonances and cell morphological changes during A549 cell adhesion in the CPALNS4c chip. (**a**) The images show cell morphological changes over time during cell adhesion. The area inside the red circle is the effective area for resonance detection. (**b**) The cell coverage percentage was plotted against time, and the time constant of cell adhesion kinetics was calculated by curve fitting. (**c**) The intensity spectra showed a redshift of Fano resonance spectra over time during cell adhesion. The d*A* is shown for a unique segment of the spectral band (600–650 nm). (**d**) The dynamics of cell adhesion were plotted as d*A* over time, and the time constant of the cell adhesion reaction was calculated by curve fitting with one-phase associated equation. (**e**) The time constants of cell adhesion were calculated from the dip shift, peak shift and d*A* kinetic curves, n = 5. Statistical significance was evaluated by a paired *t*-test. There is no significant difference between the peak shift and d*A* groups.

Both phase-contrast images and the Fano resonance spectra can be simultaneously obtained in our CAAS. We first analyzed the cellular morphology changes in a lung cancer cell line, A549. Figure 4a shows that the round cells attached to the CPALNS biosensor surface became flattened over time. We identified the Fano resonance effective detection area (the red circles) by scanning the field-of-view of the phase-contrast microscope with a pinhole (opening diameter 15 μm, data not shown). The cell morphologies and Fano resonance inside the red circle, which contained approximately ten cells, were used for cell adhesion studies. The time constant of A549 adhesion process was determined by curve fitting the measured cell coverage changes (Fig. 4b). Meanwhile, the Fano resonance spectrum showed a gradual redshift over time, which stopped at about 60 min after cell seeding (Fig. 4c). The dynamic change in the cell-sensor contact area was quantified as d*A* of the Fano resonance spectral shift, a parameter that was also used in our previous studies^23,35^. The time constant for the cell adhesion obtained by the cell coverage calculation was similar to the constant obtained from the d*A* change calculation (39 min and 37 min, respectively) (Fig. 4b,d). Yashunsky *et al*. reported a system to simultaneously collect bright-field cell images and SPR signals in a cell adhesion assay^38^. However, the cell images are not clear, and it is difficult to identify the edges of individual cells, especially flattened cells. The phase-contrast cell images in our study provide a significant improvement in the image resolution compared to the bright-field images. Therefore, the cell adhesion may be accurately analyzed using from our CAAS. Moreover, within the first 10 min of the assay, the d*A* analysis showed a significant signal change, while the cell coverage did not. This difference in d*A* change indicates that small changes in the initial cell-surface contact can be distinguished by plasmonic signal sensing but not by analysis of cell morphology. This suggest the importance to incorporate both imaging and SPR methods for detailed cell adhesion analysis.

We further assessed the adhesion process of A549 using the resonance dip and peak induced by the cells on the CPALNS4c chips. As shown in Fig. S3a,b, the resonance dip shifts faster than the peak in A549 cell adhesion tests. Additionally, the time constant calculated by the shifts of resonance dip is shorter than both the constants by the peak and d*A*. These results support the notion that the cell adhesion processes detected by the Fano resonance dip and peak are different. In Fig. 4e, no significant difference is shown between the constants calculated by the shifts of resonance peak and by d*A*, indicating that the cell adhesion responses analyzed by these two parameters tend to be identical. Therefore, we conclude that the resonance dip shift and peak shift/d*A* can be used to monitor short- and long-range cellular changes, respectively, in our cell adhesion test.

### Assessment of cell adhesion by fano resonance changes using the GOALNS25c biosensor chip

**Figure 5:**
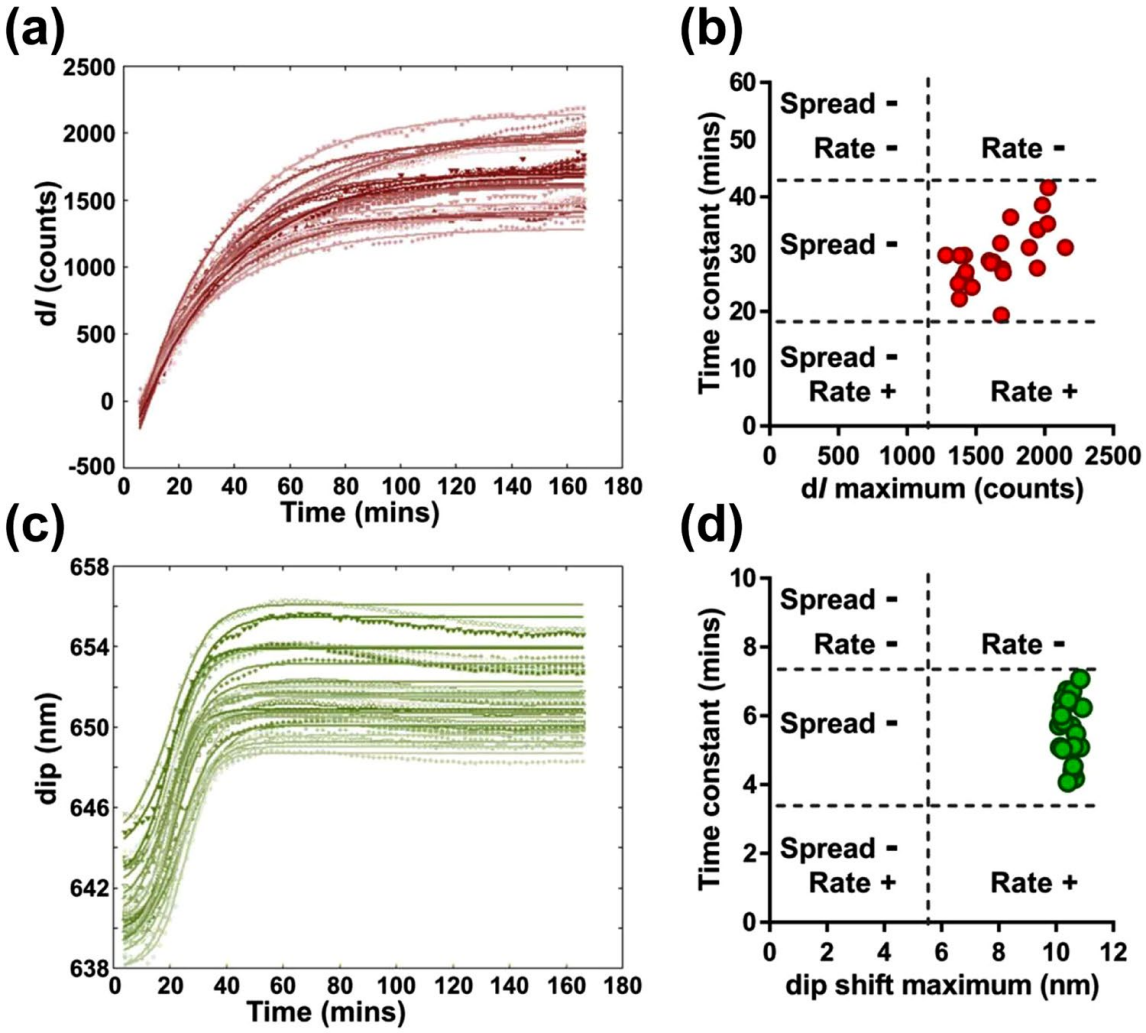
Assessment of MDCK cell adhesion by Fano resonance changes measured by the GOALNS25c chip. The cell adhesion kinetic curves were plotted using (**a**) d*I* and (**c**) dip wavelengths. Dots represent data, solid lines are the fitted curves, n = 24. The time constants (by d*I* and dip shift) were calculated by equation fitting. The profile of cell adhesion was plotted as (**b**) the d*I* time constant versus the d*I* maximum, and (**d**) the dip shift time constant versus the dip shift maximum. Cell types were classified into six categories based on the extension of spread and the adhesion rate. The dots in (**b**,**d**) represent the MDCK cell populations with normal spread and adhesion rate.

As mentioned previously, the spectral shape of the GOALNS biosensor is SPR-dominant, which means that the dip may be used for monitoring short-range cellular changes. Previously we have detected biomolecular changes by spectra intensity changes in Fano resonance using a AuNS sensor^25^. In this work, the adhesion-induced Fano resonance dip shift and intensity changes of d*I* in the GOALNS25c chip were used to assess adhesion of a normal kidney epithelial cell line, Madin-Darby Canine Kidney (MDCK) cells. Detailed analysis methods are described in the Supplementary Materials and Fig. S4. As shown in Fig. S5a, during the cell adhesion test, MDCK cells stopped spreading at 90 min after cell seeding. Moreover, morphological differences were not readily observed at 90 and 120 minutes during the test. The image result matches the cell adhesion measurement with the GOALNS25c chip in Fig. S5b, in which the spectra at 90 and 120 mins are almost identical. The temporal changes in d*I* (Fig. 5a) and the resonance dip shift (Fig. 5c) were plotted and fitted to calculate the time constants of MDCK cell adhesion kinetics using a cell adhesion model reported earlier^39^.

A highly metastatic melanoma cell line, A375 cells, was used to test the performance of GOALNS25c chip. As shown in Fig. S6a–c, the time constants of A375 cell adhesion can be obtained from the Fano resonance dip shift, the peak shift and the d*I* changes along time. There was no difference between the time constants calculated by the peak shift and by the d*I* (Fig. S6d), suggesting that both parameters can be used to monitor long-range cellular changes by the GOALNS biosensor. However, the variation in the time constant analyzed by the d*I* was smaller than that from the resonance peak shift calculation. Therefore, all subsequent cell adhesion processes were evaluated by recording and analyzing the resonance dip and the d*I* with the GOALNS25c chip.

When the process of cell adhesion progresses to late stages, the Fano resonance sensing response stopped, and no more spectral changes occurred. Therefore, the maximum value of the d*I* and the maximum shift of resonance dip can be used as a final readout for cell adhesion. By plotting the time constant versus dip shift maximum or d*I* maximum, the adhesion characteristics of MDCK cells could be classified into six categories (Fig. 5b,d) based on the extension of spread and the adhesion rate. The extension of spread is categorized into two states: normal and low (marked with “−” in Fig. 5) while the adhesion rate is categorized into three states: fast (marked as “+” in Fig. 5), normal and slow.

**Figure 6:**
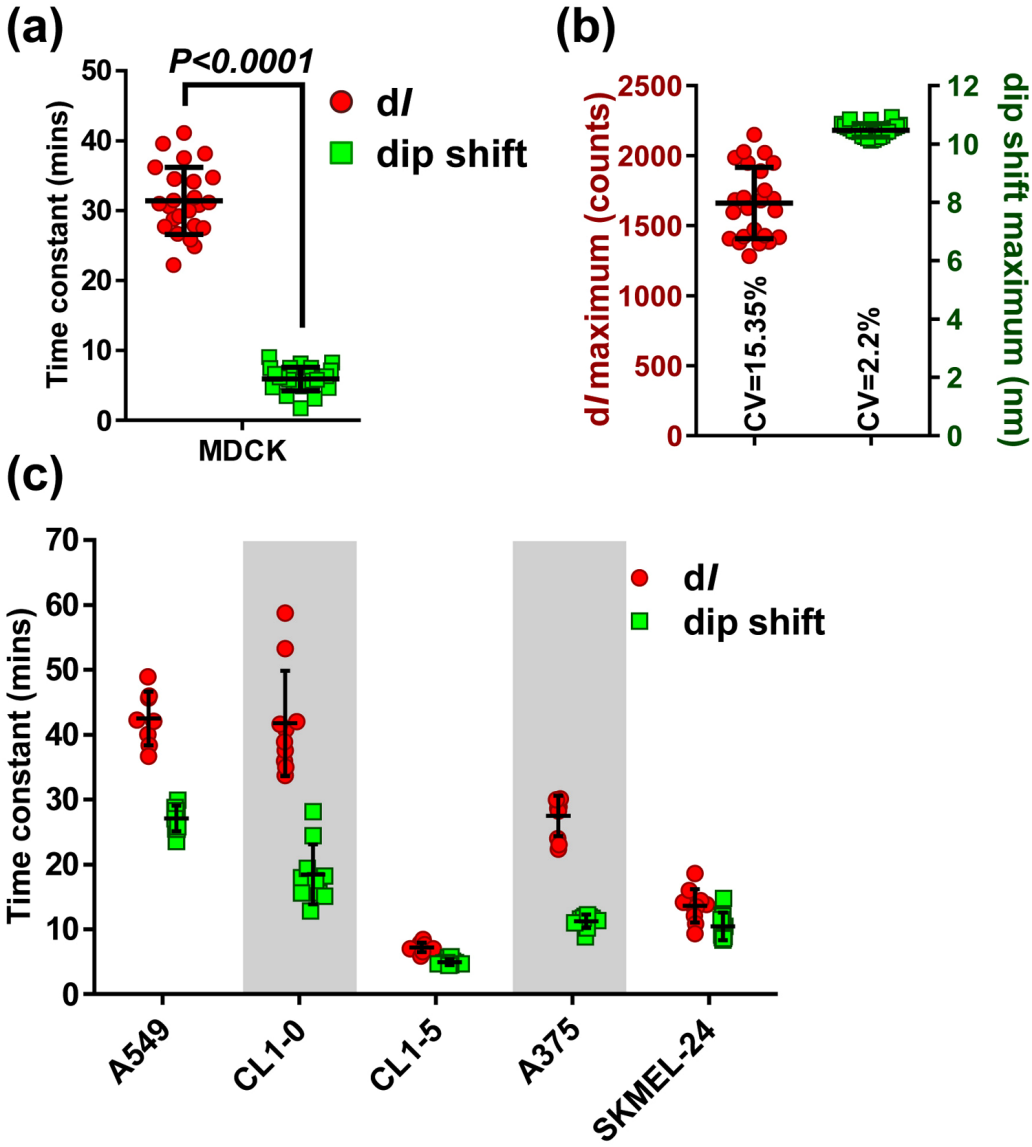
The cell adhesion rates of normal and cancer cells. (**a**) The time constants of MDCK cell adhesion were obtained from curve fitting of the d*I* and the dip shift. (**b**) Coefficients of variation for the d*I* maximum and the dip shift maximum from cell adhesion assessment with the GOALNS25c chip, n = 24. (**c**) The time constants of cancer cells were calculated by d*I* and dip shift curve fitting. There were significant differences between d*I*- and dip shift-calculated time constants in all cancer cell groups, n = 7–10 for each group; lung cancer cells: CL1-0, CL1-5 and A549; melanoma cells: A375 and SKMEL-24. All data are presented as mean ± S.D.

Figure 6a shows that the time constant analyzed by the d*I* and the resonance dip changes of MDCK cells were 31.4 min and 5.9 min, respectively. As expected, the cell adhesion rate was faster when calculated from the resonance dip shift. The result corresponds well to the fact that cell adhesion starts with rapid interaction in region that is in proximity to the surface. Moreover, the extent of spreading and the total cell numbers also may affect the d*I* maximum and the dip shift maximum. Shown in Fig. 6b, the coefficient of variation for the dip shift maximum was significantly smaller than that for the d*I* maximum, indicating that the dip shift maximum is better for determining the final extent of cell spread in CAAS using the GOALNS25c chip.

### Comparing adhesion of cancer cells using the GOALNS25c biosensor chip

In order to explore the correlation between metastatic potency and cell adhesion, three lung cancer cell lines (CL1-0, CL1-5 and A549 cells) and two melanoma cell lines (A375 and SKMEL-24 cells) were assessed using the GOALNS25c chip.

As shown in Fig. 6c, time constant calculated by the dip shift was shorter than that by the d*I* in all tested cancer cell lines. Interestingly, for both types of calculation, the higher metastatic CL1-5 cells showed shorter time constants than the lower metastatic CL1-0 cells. This result indicates that the CL1-5 cells have higher cell adhesion rate than the CL1-0 cells. However, the cell adhesion rate of CL1-0 and A549 cells were similar according to our measurements.

In the adhesion test using GOALNS sensor, the long- and short-range cellular response in the longitudinal direction can be respectively analyzed by the d*I* changes and the dip shifts. The dynamic change in the longitudinal direction might help us to evaluate the cell adhesion processes such as the dynamic cellular properties or thickness change.

In our result, the time constant ratio was similar for A549 cells and CL1-5 cells, but different from that for CL1-0 cells (A549: 1.56, CL1-5:1.46, CL1-0: 2.26). According to a previous report, metastatic potency based on invasiveness assay is the highest for CL1-5 cells, moderate for A549 cells and the lowest for CL1-0 cells^40^. The value of the ratio may correlate with the metastatic potency of lung cancer cells in our test.

For the melanoma cells, it has been reported that the metastatic potency of A375 cells is similar to that of SKMEL-24 cells^41^. In our results, the SKMEL-24 showed shorter d*I* time constant than the A375 cells but the time constants by the dip shift are similar. These results shows that short-range detection indicates similar adhesion rate but long-range detection indicates differently. Additionally, the ratio of the time constant by the two types of calculation was also different in A375 and SKMEL-24 cells (A375: 2.44 and SKMEL-24: 1.3). Notably, the correlation between time constant ratio of d*I* to dip shift and metastatic potency of melanoma cells is not as clear as for the lung cancer cells and needs further investigation. Nevertheless, the dip shift time constant can be a potential parameter for determination of metastatic potency in cancer cells.

The time constant ratio may indicates final cell thickness of the adherent cells. The thickness of adherent MDCK cells was reported to be 8–10 μm and A549 cells were reported to be approximately 2 μm^42–44^. The time constant ratio of MDCK cells and A549 cells was 5.19 and 1.34, respectively, suggesting that smaller time constant ratio may correspond to thinner adherent cells. It is reasonable that thicker cells would require more time to form a thicker monolayer. Thus, the time constant ratio of d*I* to dip shift might be used for estimating the thickness of the adherent cells.

### The effects of inhibitors on adhesion measurements by the GOALNS25c biosensor chip

We next tested whether small molecules that alter cell adhesion activity could potentially be identified by our biosensor chips. We used the time constant calculated by resonance dip shift to compare the adhesion rate of different kinds of cancer cells in the GOALNS25c chip. Additionally, dot plots of the time constant versus the dip shift maximum further elucidated the characteristics of cell adhesion under various stimuli. Using these analyses, we tested the effect of an adhesion inhibitor on cancer cell adhesion rates using the GOALNS25c chip. Focal adhesion formation is known to be controlled by FAK^9^, and the FAK inhibitor, FAKi-14, has been reported to suppress the cell adhesion in human pancreatic cancer^45^. Thus, we treated four types of cancer cells with FAKi-14 and assessed the effect of the drug on the cell adhesion.

**Figure 7:**
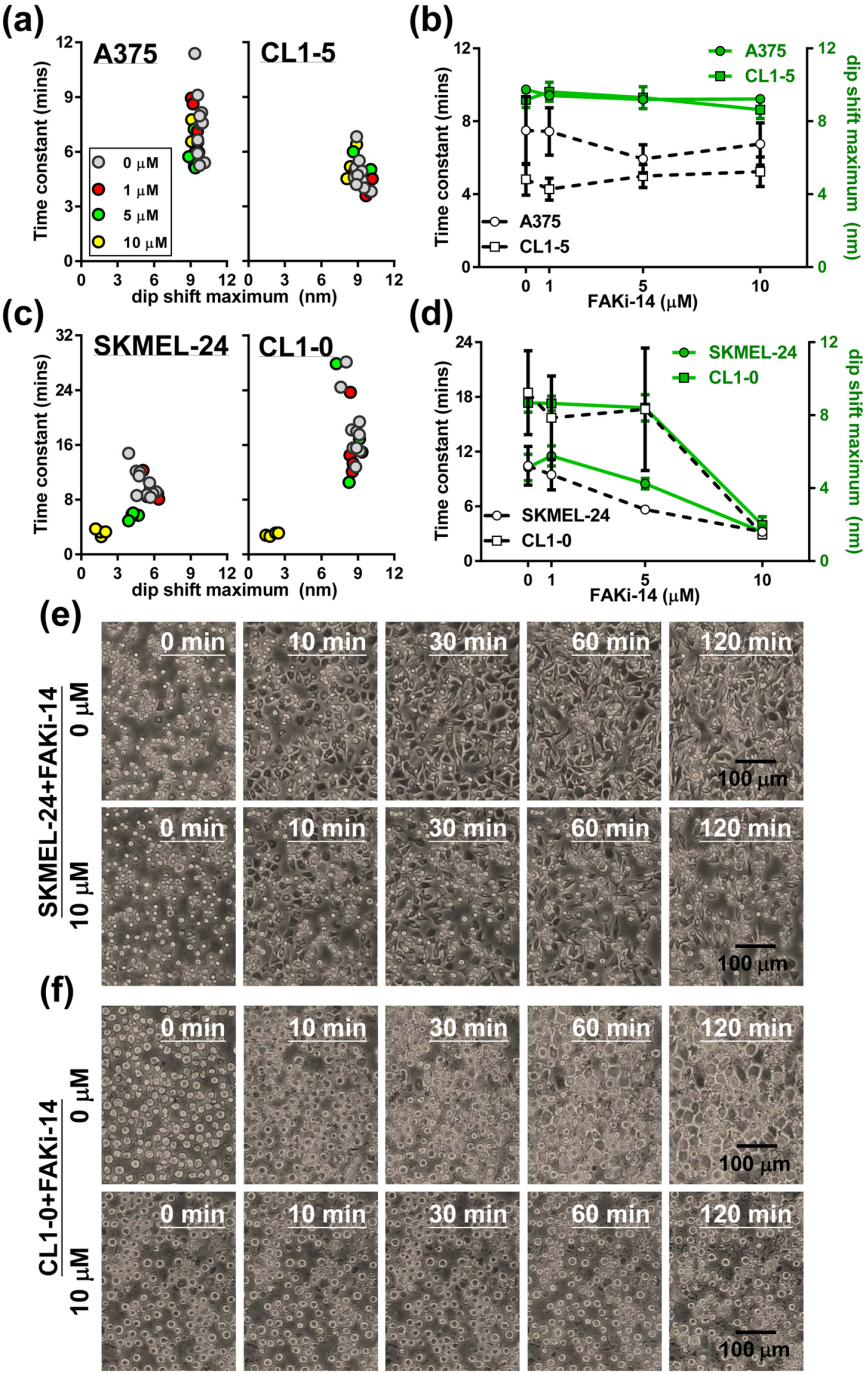
The effects of a cell adhesion inhibitor on the adhesion of melanoma and lung cancer cells. (**a,c**) Adhesion of cancer cells in response to different concentrations of cell adhesion inhibitor, FAKi-14. The plots show dip shift time constant versus dip shift maximum. (**b,d**) A dose-dependent effect of the cell adhesion inhibitor was observed for dip shift time constant and dip shift maximum. Data are presented as mean ± S.D. The images show (**e**) SKMEL-24 and (**f**) CL1-0 cell morphological changes over time during cell adhesion with or without cell adhesion inhibitor present.

As shown in Fig. 7, two cancer cell lines, A375 and CL1-5 were insensitive to the inhibitor, while the other two lines, SKMEL-24 and CL1-0 were responsive to the drug. In Fig. 7a, it shows that the responses of the inhibitor-treated cells could not be distinguished from those of the non-treated cells. On the other hand, one or two subgroups appear in the left-lower corner of the plot for CL1-0 and SKMEL-24 cells (Fig. 7c). The appearance of these subgroups suggests that these cells were significantly affected by FAKi-14 treatment and showed a poor extension of spreading. The spreading of the FAKi-treated cells were interrupted and reached steady state earlier than the normal cell. Moreover, the incomplete and interrupted adhesion compared to the normal cells result in an increased adhesion rate (i.e. lower time constant). Additionally, a dose-dependency was observed in FAKi-14-treated SKMEL-24 cells, wherein both the dip shift time constant and the dip shift maximum decreased with increasing concentration of the inhibitor (Fig. 7d). While the cell adhesion rates of A375 and CL1-5 cells were not affected by drug concentrations as high as 10 μM (Fig. 7b), the cell adhesion rate of SKMEL-24 and CL1-0 were significantly increased by the drug at a concentration of 10 μM. The suppression of the cell spreading by the inhibitor was also observed in the images of SKMEL-24 cells (Fig. 7e) and CL1-0 cells (Fig. 7f).

To sum up, we can use two parameters (dip shift time constant and dip shift maximum) to evaluate the inhibitory effects of a compound on cell adhesion using GOALNS biosensor. As shown in Fig. 7b,d, the dose-dependency curves derived from the dip shift time constant and the dip shift maximum show a similar trend. Despite the higher variation in the dip shift time constant, we consider both parameters can be used to assess cell adhesion response. It is interesting that the two melanoma cell lines exhibit similar metastatic potencies but differ in their sensitivities to FAKi-14. In these lines, it is possible that the FAK-activated adhesion pathway differs in terms of kinase activity or expression levels. Further experiments may be performed using extracellular matrix proteins, such as fibronectin, collagen and laminin to specifically activate the FAK adhesion pathway during the cell adhesion test. Nevertheless, in this report, we clearly show that the GOALNS25 chip is able to detect the effects of inhibitors on cell adhesion.

## Conclusion

In this study, we built a new system for simultaneous collection of cell morphology and Fano resonance signals to monitor cell adhesion. In this system, sensitive double-layer Al nanoslit biosensors were used to evaluate the adhesion of normal and cancer cells. The experimental spectra were collected, and the adhesion time constants were computed using d*A*, d*I*, peak shift and dip shift of the Fano resonance signals, which were induced by the binding of cells to the nanoslit. By using the Al nanoslit-based biosensor chip, we were able to sense both short- and long-range changes during the cell adhesion process. The time constants calculated from dip shifts and d*I* of the Fano resonance allowed us to compare the cell adhesion rates among normal and cancer cells. Additionally, we could examine certain features of cell adhesion using the d*I*- and dip shift time constants, d*I* maximum and dip shift maximum. Since the detection depth is different for d*I* and resonance dip, the time constant ratio of d*I* to dip shift was found to have some potential for estimating the thickness of adherent cells and the metastatic potencies of lung cancer cells. Assessment of cell adhesion by the resonance dip change is more reliable than the d*I* change because the resonance intensity change can be affected by damage to the nanoslit structure and cell scattering effects in the test. We successfully determined the adhesion rate of cancer cells as well as their response to an adhesion inhibitor with the Al nanoslit-based biosensor chip. Moreover, we are able to rapidly produce a large number of nanoslit-based biosensors at low-cost. Therefore, the Al nanoslit-based plasmonic biosensing chips show great potential for drug screening applications in the future.

## Materials and Methods

### The optical setup of cell adhesion assessment system

The setup for the CAAS is shown in Fig. 1a. The system is based on an inverted phase-contrast microscope (IX71, Olympus) with an integrated motorized stage, proportional integral derivative (PID; TTM-J40-R-AB, JETEC Electronics Co.)-controlled transparent heater, spectrometer (V2000, Ocean Optics) and digital camera (EOS-D60, Canon). The temperature of the biosensor chip was measured by a K-type thermocouple (TPK-02A, TECPEL) clipped between the chip and the indium tin oxide glass (ITO glass, Part No. 300739, Merck) transparent heater. Images of cells were obtained with a digital camera. The light path configuration shown in Fig. 1b allowed us to simultaneously collect spectrum signals and phase-contrast images.

### Fabrication of SPR biosensors and cell adhesion sensing chips

Two types of novel nanostructure sensors, CPALNS and GOALNS, were fabricated and used for cell adhesion assessment. The nanostructural schematics of the biosensors are shown in Fig. 1c,d. The scanning electron microscope images of the capped and the grooved aluminum nanoslits are shown in Fig. S7. The nanostructure induces light in transverse and longitudinal direction. The composition diagrams of cell adhesion sensing chips are shown in Fig. 2a,d. Fabrication of the nanoslit plastic substrate was described previously^46^. Briefly, a 130-nm-thick diluted ZEP-520 resist (ZEP-520, Zeon Corp, Tokyo, Japan) was spin-coated onto a 4-inch silicon substrate. Periodic nano-groove arrays were fabricated in the resist using an electron beam drawing system. The period and width of the nano-groove arrays were 470 nm and 60 nm, respectively. The resist patterns were then coated with gold using a sputter and electroformed with Ni and Co to produce a ridged nanoslit metal mold (RNMD). The RNMD was then used in the production of nano-groove array plates by compression-injection molding. A picture of the nano-groove array plastic plate is shown in Fig. 2e. A grooved nanoslit metal mold (GNMD) was obtained by direct electroforming with the RNMD. The GNMD was used to produce the nano-ridge arrays by hot-embossing nanoimprinting. A picture of the nano-ridge array plastic film is shown in Fig. 2b. After depositing Al on the nanoslit array plastics with a thermal evaporator, we obtained the CPALNS and the GOALNS biosensors. A four-chamber cell adhesion assessment chip, called the CPALNS4c chip, was designed and fabricated using PMMA, double-sided tape and the CPALNS biosensor (Fig. 2a,c). A 25-chamber cell adhesion assessment chip, called the GOALNS25c chip, was assembled by PMMA, double-sided tape and the GOALNS biosensor (Fig. 2d,f). The volumes of the cell culture chambers in the CPALNS4c and the GOALNS25c chips were 54.7 mm^3^ (6 × 6 × 1.52 mm) and 43.2 mm^3^ (4.1 × 4.1 × 2.57 mm), respectively. All of the acrylic sheets and the double-sided tapes were fabricated by laser ablation using a CO_2_ laser scriber (V-2000, LTT group, Taiwan), according to design patterns drawn in AutoCAD (Autodesk). Details of the micro-channel fabrication procedures using a CO_2_ laser were provided in a previous work^47,48^. All components of the chip were disinfected by 30 min UV irradiation before assembly. The completed sensor chip assembly was then treated with oxygen plasma for 120 s at 200 W. Complete culture medium was incubated in a 5% CO_2_ incubator at 37 °C overnight prior to injection into the culture chamber. Before the cell adhesion test, all chambers were washed once with distilled water and air dried for 20 min in a laminar flow cell culture hood.

### Cell adhesion assessment

In order to enhance cell adhesion of the cancer cells, the biochip surface was coated with complete culture medium before cell injection. Approximately 90 μl (in the CPALNS4c chip) and 45 μl (in the GOALNS25c chip) of cells (5 × 10^5^ cells/mL) were injected or loaded into the chamber of the cell adhesion sensing chip. The chip was then incubated on a computer-controlled motorized stage. The optical wavelength change was recorded by a spectrophotometer every two minutes during the cell adhesion assessment; the total experimental period was 2–4 h depending on the cancer cell type. Cell adhesion kinetic curves were expressed as the total integrated intensity changes and the specific spectra shift over time.

### Data analysis

Fano resonance changes induced by cell attachment and spreading were analyzed using the d*A* method^22,23,46^, according to the equation:

$${\rm{d}}A={\sum }_{\lambda =650}^{\lambda =600}|\frac{I(\lambda )-{I}_{0}(\lambda )}{{I}_{s}(\lambda )}|$$

where d*A* is the SPR response, *I*_0_ (λ) is the intensity of the referenced spectrum (time = 0), *I* (λ) is the intensity of the spectrum at the indicated time point and *I*_s_ (λ) is the intensity of a constant wavelength (resonance substrate mode), which served as an internal control. The spectral integration region was from 600 to 650 nm, in which Fano resonance occurred at the Al/medium interface. The cell adhesion response curve was plotted as d*A* over time, and the curves were fitted with an equation (one-phase association) in Prism 6 software (GraphPad). The time constant was obtained from the fitted equation. The d*I* was calculated as:

$${\rm{d}}I={I}_{\max \,\lambda =645}^{\lambda =620}\,[{I}_{i}(\lambda )-{I}_{0}(\lambda )]$$

where the d*I* is the cell adhesion response, *I*_0_ (λ) is the intensity of the spectral band from 620 to 645 nm at time 0, *I*_*i*_ (λ) is the intensity of the spectral band from 620 to 645 nm at an indicated time point, *I*_*max*_ is the intensity peak of differential intensity spectral. The resonance dip shift, peak shift and the intensity peak of the differential spectra were obtained by fitting the data with a Gaussian distribution. The cell adhesion kinetic curves were plotted as dip shift, peak shift and d*I* over time and fit with the logistic equation for cell spreading to calculate time constants^39^.

## Supplementary information

Supplementary information
