# Supplementary material for: Simultaneous assessment of cell morphology and adhesion using aluminum nanoslit-based plasmonic biosensing chips
Source: Sci Rep. 2019 May 10;9:7204. doi: 10.1038/s41598-019-43442-w (PMC6510726; doi:10.1038/s41598-019-43442-w)
Supplement: Supplementary file 1 — Supplementary information [file 41598_2019_43442_MOESM1_ESM.docx]

Title: Simultaneous assessment of cell morphology and adhesion using aluminum nanoslit-based plasmonic biosensing chips

Authors:

Hsien-San Hou,^a^

Kuang-Li Lee,^a^

Chen-Hung Wang,^a^

Tung-Han Hsieh,^a^

Juan-Jie Sun,^a^

Pei-Kuen Wei, ^a,b^

Ji-Yen Cheng ^a,b,c,d,*^

*^a^Research Center for Applied Sciences, Academia Sinica, Taipei 11529, Taiwan*

*^b^Institute of Biophotonics, National Yang-Ming University, Taipei 11221, Taiwan*

*^c^Department of Mechanical and Mechatronic Engineering, National Taiwan Ocean University, Keelung 20224, Taiwan*

*^d^College of Engineering, Chang Gung University, Taoyuan 33302, Taiwan*

**Supplementary Materials**

**Materials and Methods**

***Reagents and cell culture***

Lung cancer cell lines CL1-5 and CL1-0 were obtained from Prof. Pan-Chyr Yang [^1^](#_ENREF_1). Melanoma cell lines A375 and SKMEL-24 were kindly provided by Dr. Chung-Hsing Chang, Institute of Medical Sciences, Tzu Chi University. The cells were cultured in complete medium composed of Dulbecco’s Modiﬁed Eagle’s medium (DMEM, Gibco, USA) and 10% fetal bovine serum (FBS, Invitrogen, USA). Lung cancer cell line A549 was purchased from ATCC. The cells were cultured in the complete medium composed of Ham's F-12K (Kaighn's) medium (F-12K, Gibco, USA) and 10% fetal bovine serum (FBS, Invitrogen, USA). The MDCK cells were kindly provided by Dr. Tang Tang, Institute of Biomedical Sciences, Academia Sinica, Taiwan. The cells were cultured in the complete medium composed of Minimum Essential Media (MEM, Gibco, USA) and 10% fetal bovine serum (FBS, Invitrogen, USA). For regular maintenance, the cells were incubated at 37^o^C in a 5% CO_2_ atmosphere and passaged every 3 to 4 days. Cell adhesion experiments were performed on cells within 25 passages after receipt. Cells without mycoplasma infection were used in this study. The effects of FAK Inhibitor 14 (FAKi-14, Sigma-Merck, Germany) were investigated on the cell adhesion of CL1-0, CL1-5, A375 and SKMEL-24 cells.

***Analysis methods for cell adhesion-induced Fano resonance changes in GOALNS25c chip***

Dip shift and intensity change of the Fano resonance in the GOALNS biosensor were used to analyze MDCK cell adhesion. The wavelength of the resonance dip and the differential spectral peak intensity (d*I*) (Figure S4a) were obtained by fitting the spectrum curve with a Gaussian equation. As shown in Figure S4b, the resonance dip appears at around 640 nm at time-point zero. The Fano resonance dip showed a redshift at 60 min after cell seeding. In the intensity change analysis, a differential spectral peak was observed during the cell sensing. As shown in Figure S4c, the differential spectrum was calculated by subtracting the intensity spectra from the initial spectrum (Figure S4c). Meanwhile, the differential spectral peak showed a significant increase in intensity at certain wavelengths (Figure S4c).

**References**

1 Chu, Y. W. *et al.* Selection of invasive and metastatic subpopulations from a human lung adenocarcinoma cell line. *American journal of respiratory cell and molecular biology* **17**, 353-360, doi:10.1165/ajrcmb.17.3.2837 (1997).

**Figures**

**
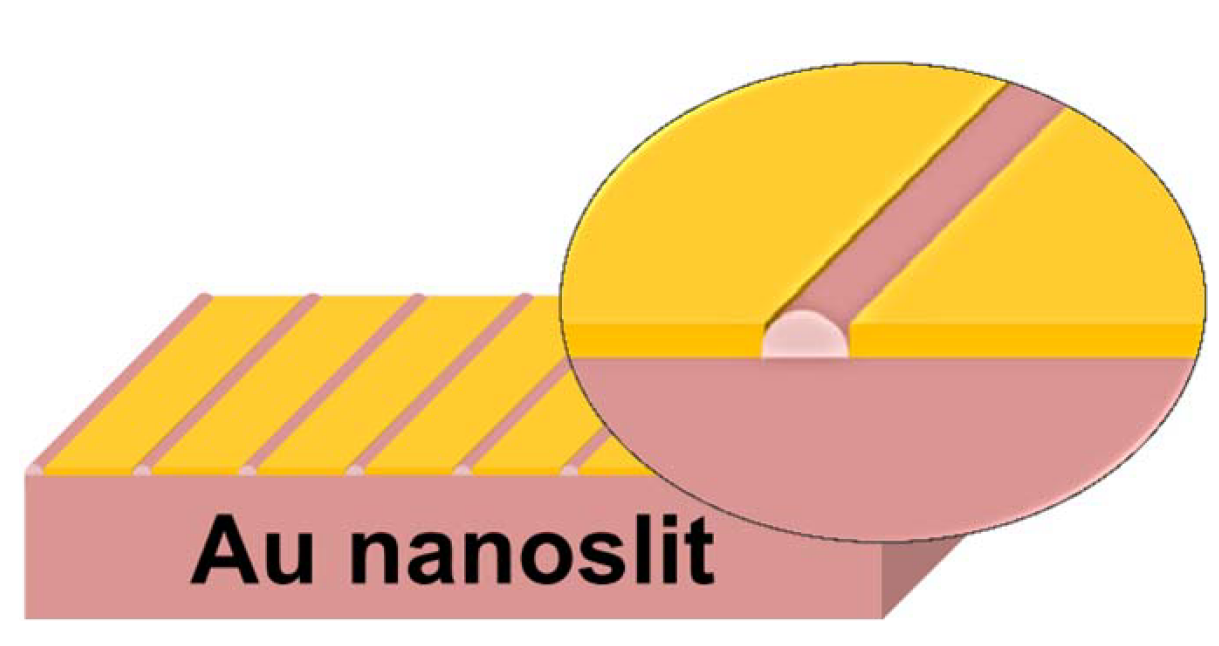
**

Figure S1. **Schematic diagram of the monolayer AuNS sensor**. Yellow layer indicates the deposition of gold on the nanoimprinted polycarbonate (PC) film (pink).


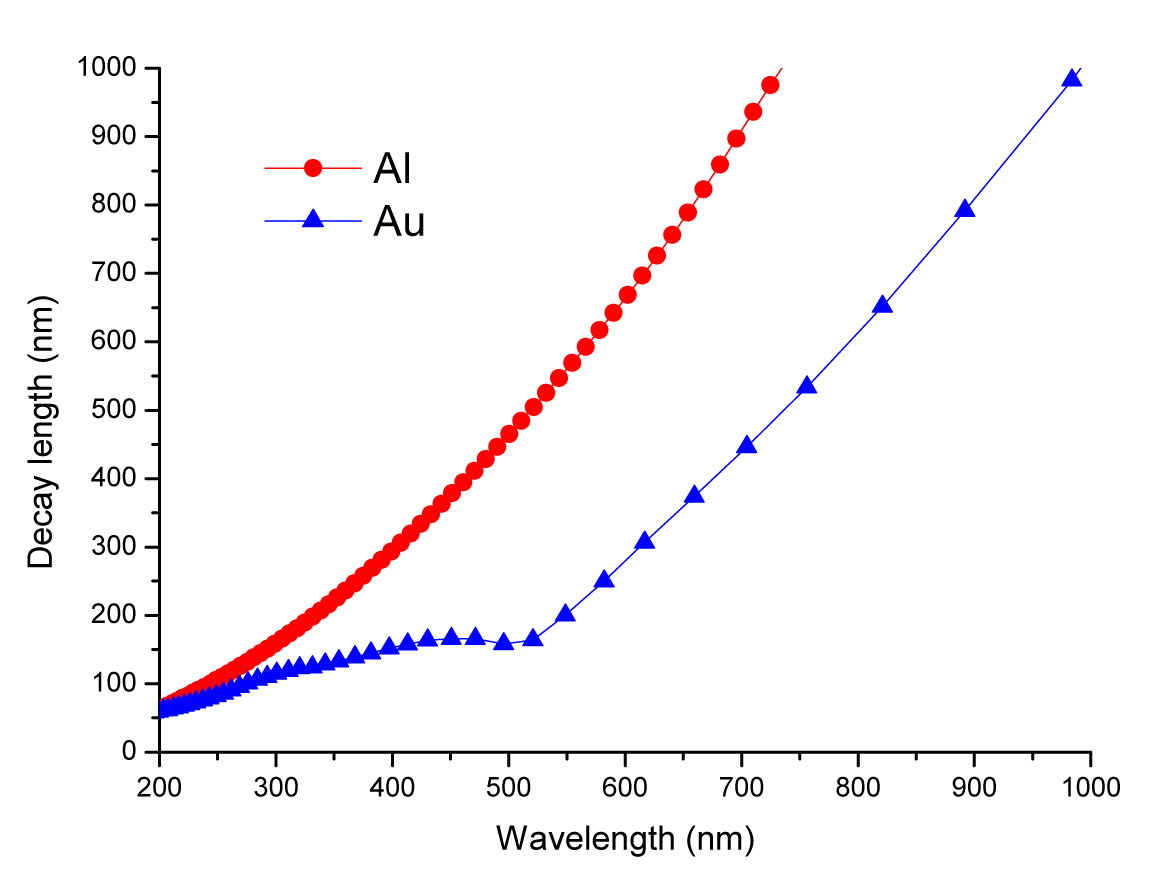


Figure S2. The calculated decay lengths at indicated SPR dip wavelength for Al and Au film.


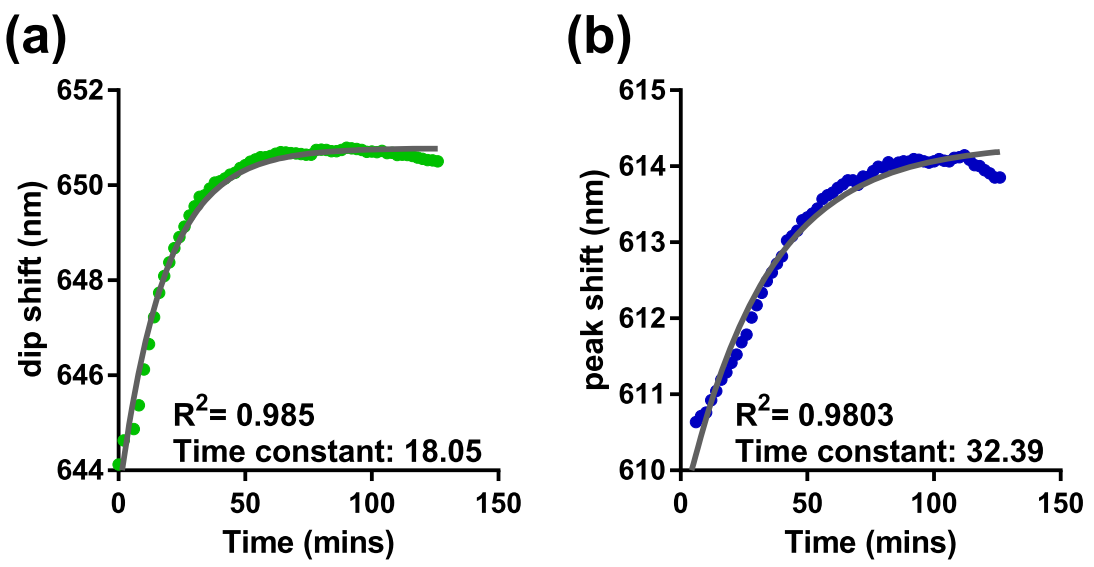


Figure S3. **The Fano resonance changes during A549 cell adhesion in CPALNS4c chip.** The dynamics of A549 cell adhesion were plotted as (a) resonance dip shift and (b) peak shift over time. Dots represent data, solid lines are the fitted curves.


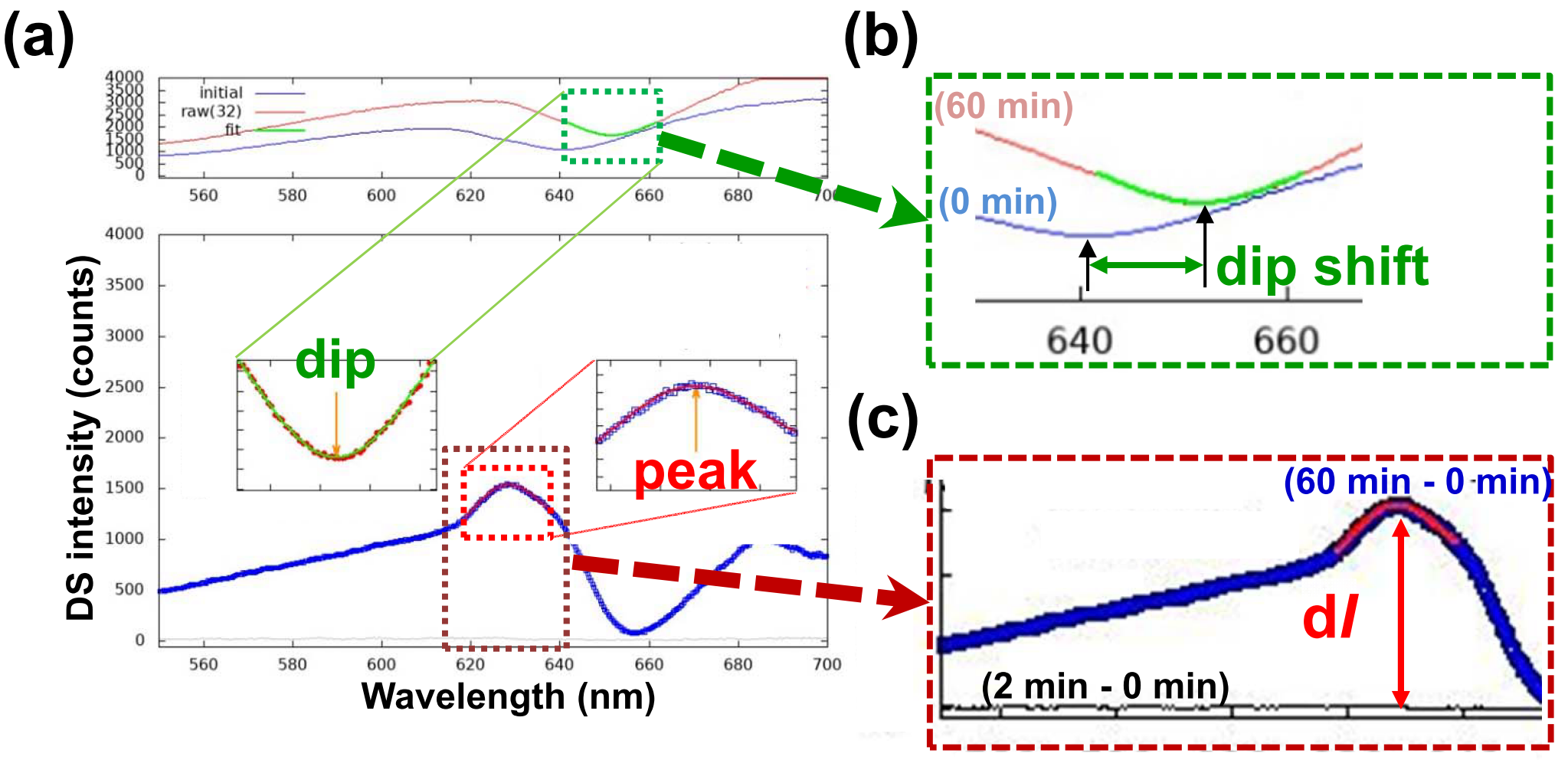


Figure S4. **Analysis of cell adhesion-induced Fano resonance changes in GOALNS25c chip.** (a) The SPR intensity spectrum of the 60 min MDCK cell adhesion assessment. The intensity spectrum showed (b) the dip shift and (c) the d*I* during cell adhesion. The wavelength of the resonance dip and the intensity of the differential spectral peak (DSP) were obtained by Gaussian fitting.


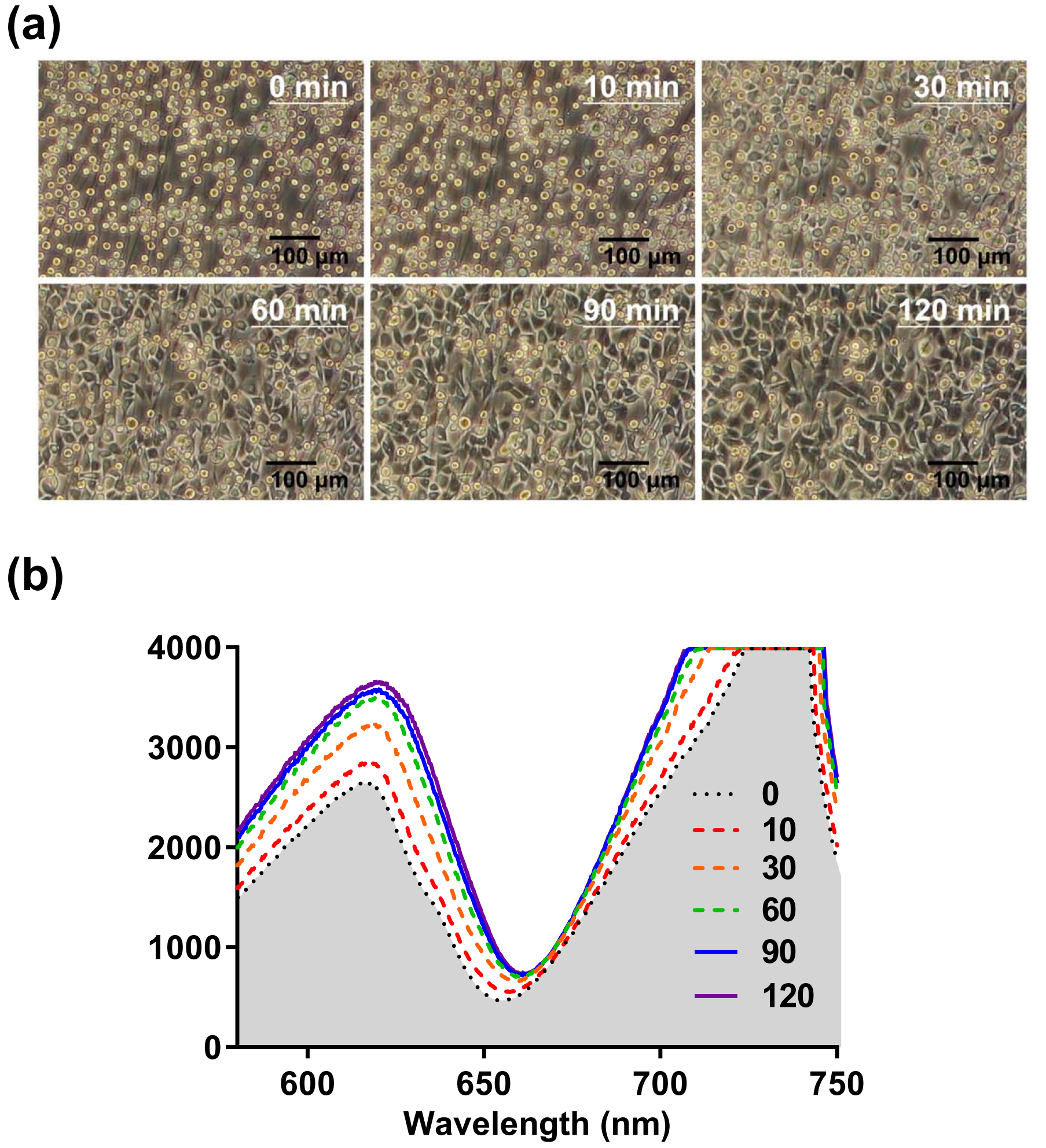


Figure S5. **The Fano resonances and cell morphological changes during MDCK cell adhesion in the GOALNS25c chip**. (a) The images of the cell morphological changes over time during cell adhesion. (b) The intensity spectrum shifts of the Fano resonance induced by cell adhesion process at 0-120 mins after cell seeding.


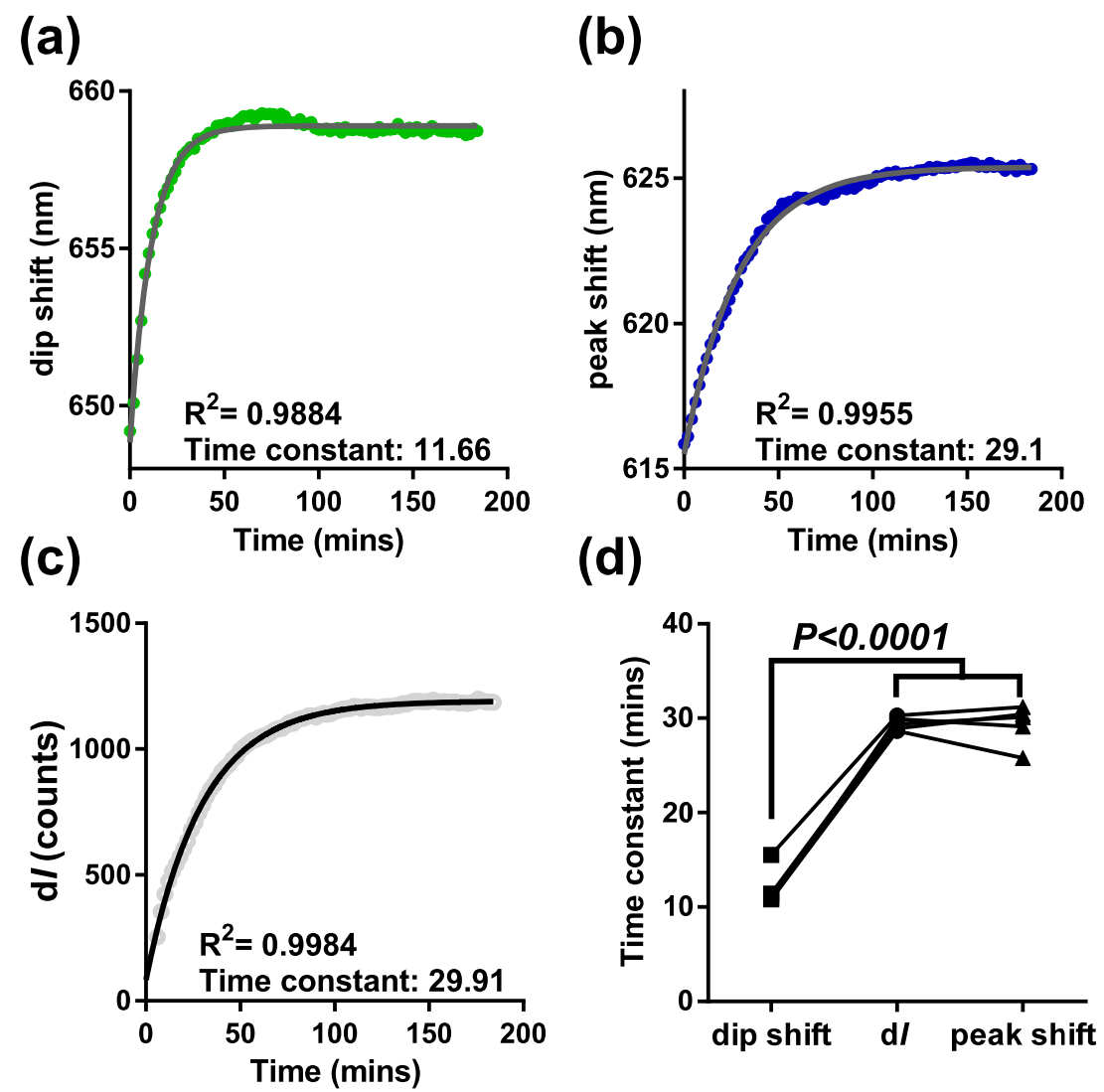


Figure S6. **Fano resonance change during A375 cell adhesion in GOALNS25c chip.** The dynamics of A375 cell adhesion were plotted as (a) resonance dip shift, (b) peak shift and (c) d*I* over time. Dots represent data, solid lines are the fitted curves. (d) The time constants were calculated from resonance dip shift, peak shift and d*I* curve fitting, n = 4. Statistical significance was analyzed by paired *t*-test. There was no significant difference between the d*I* and peak shift groups.


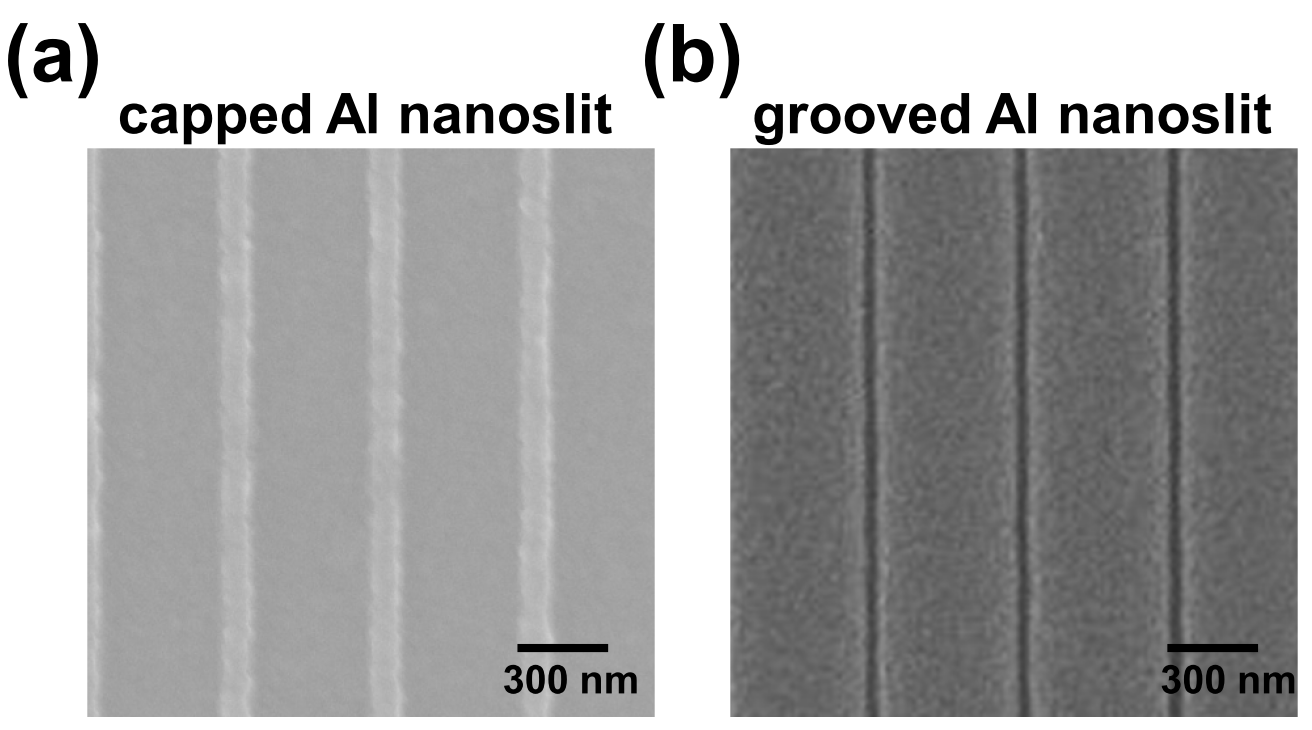


Figure S7. The scanning electron microscope (SEM) images of (a) the capped and (b) the grooved aluminum nanoslit.
